# Exploring the potential of biosurfactants produced by fungi found in soil contaminated with petrochemical wastes

**DOI:** 10.1038/s41598-024-75865-5

**Published:** 2024-10-28

**Authors:** Yehia A.-G. Mahmoud, Yasser H. El-Halmouch, Ebtsam E. Nasr, Yassin M. Al-Sodany, Baher A. El-Nogoumy, Sameh S. Ali

**Affiliations:** 1 Botany Department, Faculty of Science, Tanta University, Tanta, 31527 Egypt; 2 Department of Botany and Microbiology, Faculty of Science, Kafrelsheikh University, Kafr Elsheikh, 33511 Egypt

**Keywords:** Biosurfactant, Bioremediation, Hydrocarbon oils, Bioprocess, Petrochemical wastes, Environmental safety, Biotechnology, Microbiology, Environmental sciences

## Abstract

Biosurfactants are a diverse group of compounds derived from microorganisms, possessing various structures and applications. The current study was seeking to isolate and identify a new biosurfactant-producing fungus from soil contaminated with petrochemical waste. The bioprocess conditions were optimized to maximize biosurfactant production for *Aspergillus carneus* OQ152507 using a glucose peptone culture medium with a pH of 7.0 and a temperature of 35 °C. The carbon source was glucose (3%), and ammonium sulfate (0.25%) was utilized as the nitrogen source. For *Aspergillus niger* OQ195934, the optimized conditions involved a starch nitrate culture medium with a pH of 7.0 and a temperature of 30 °C. The carbon source used was sucrose (3.5%), and ammonium sulfate (0.25%) served as the nitrogen source. The phenol-H_2_SO_4_ and phosphate tests showed that the biosurfactants that were extracted did contain glycolipid and/or phospholipid molecules. They showed considerable antimicrobial activity against certain microbes. The obtained biosurfactants increased the solubility of tested polyaromatic hydrocarbons, including fluoranthene, pyrene, anthracene, and fluorine, and successfully removed the lubricating oil from contaminated soil and aqueous media surface tension reduction. Based on the obtained results, *A. carneus* and *A. niger* biosurfactants could be potential candidates for environmental oil remediation processes.

## Introduction

Biosurfactants, as amphiphilic surface-active substances, specialize in accumulating at the interface of immiscible fluids to decrease surface and interfacial tensions, thereby augmenting the surface areas of insoluble substances for enhanced mobility. The amphiphilic structure of a surfactant generally comprises a hydrophobic moiety, such as a long-chain fatty acid, α-alkyl-β-hydroxy fatty acid, or hydroxyl fatty acid, in conjunction with a hydrophilic moiety that may include carbohydrates, peptides, amino acids, phosphate groups, carboxylic acids, alcohols, or other compounds^1^. They are unique secondary metabolites synthesized non-ribosomally by specific bacteria and fungi^2^. Bacteria such as *Bacillus* sp., *Pseudomonas* sp., and *Acinetobacter* sp. are the predominant producers of biosurfactants, alongside fungi including *Trichoderma atroviride*,* Trichoderma citrinoviride*,* Trichoderma reesei*, *Trichoderma harzianum*, *Fusarium fujikuroi*,* Aspergillus niger*, and *Penicillium chrysogenum*^3,4^. The process of biosurfactants emulsifying hydrophobic substrates plays a crucial role in enhancing the availability of essential nutrients required for the development of organisms^5^.

Several recent studies have highlighted various strategies and advancements aimed at improving biosurfactant production. These strategies include isolating and identifying new efficient biosurfactant producers^6^; manipulating the genetic makeup of microorganisms^7^; optimizing nutrients for carbon sources, nitrogen sources, and other nutrients^8^; adjusting process parameters by optimizing conditions like pH, temperature, and agitation speed; and cost-effective methods for extracting and purifying biosurfactants^8^, and ensuring the sustainability of biosurfactant production^9^. Research is ongoing to tailor the properties of biosurfactants for various applications^10^. For biosurfactants to be useful in a wide range of biological situations, they need to have certain physical and chemical properties. These include the ability to lower surface tension, biodegrade oil, keep emulsions stable, recover oil efficiently, inhibit microbes, and dissolve in hydrocarbons. Biosurfactants are better than chemical surfactants in many ways, including being less harmful, breaking down more quickly, making more foam, being safe for the environment, and working well in tough conditions^11^. They are a desired class of chemicals due to their diversity, which makes them useful for a variety of industrial and biotechnological applications, such as chemistry, pharmaceutics, cosmetics, and food production^12,13^.

The objective of this study was to isolate, characterize, and identify biosurfactants produced by fungi found in soil contaminated with petrochemical wastes. It also aimed to optimize the productivity of these biosurfactants and evaluate their potential applications in areas such as oil recovery, microbial enhanced oil recovery, polyaromatic hydrocarbon solubility, and antibacterial activity. Additionally, the study aimed to provide a detailed description of the chemical structure of the identified biosurfactants. Hence, this study’s findings advance our understanding of biosurfactants, their potential applications, and provide insights into their production and chemical structure. The knowledge gained from this research has the potential to drive innovation and contribute to the development of sustainable and environmentally friendly technologies.

## Material and method

### Isolation of biosurfactant producer fungal strains

Fungi were isolated from crude oil-contaminated soil and water samples taken from Kafr El-Sheikh governorate, Egypt, and stored in sterile glass bottles and sterilized plastic bags in PDA as pure cultures. A serial dilution was prepared to 10^− 4^ using one gram of a petrochemical soil sample in a sterile tube with nine milliliters of sterilized distilled water, followed by plating 100 µl of each dilution onto Sabouraud agar medium^14^. The colonies that developed were isolated in sterile Petri dishes using the identical culture following 72 h of incubation at 29 °C. The identical culture media was employed to preserve fungal colonies in slants, which were subsequently identified by examining their macroscopic and microscopic features^15^. In this study, a total of 89 morphological different fungal isolates were obtained.

### Biosurfactant-producing fungi cultivation

The fungal isolates that were effectively carried out using potato dextrose broth culture medium supplemented with 10 ml/l sunflower seed oil were selected. The emulsification index and oil spreading test were used to evaluate the potential of biosurfactant-producing fungi. Using morphological and microscopical features, *A. carneus* and *A. niger* have been identified as the most promising fungi for biosurfactant production among all tested isolates. Then *A. carneus* and *A. niger* were cultured in Erlenmeyer flasks containing 100 ml of the liquid medium with 10 ml/l of sunflower oil. The culture medium underwent inoculation with spores of the isolates, reaching a final concentration of 1 × 10^4^ spores/ml. Subsequently, the flasks were incubated for 7 days at 29 ± 2 °C with vigorous shaking at 130 rpm. Post-incubation, the mixture was subjected to filtration using a quantitative 12.5 cm filter paper with a pore size of 28 m to eliminate fungal cells. The resulting filtrate was isolated for subsequent utilization in the evaluation of biosurfactants through oil spreading tests and determination of the emulsification index^16^.

### Fungal identification and biosurfactant production optimization

The isolates were described by their morphological characteristics (color, colony diameter, and texture), microscopic characteristics (mycelium morphology, spore color and shape, phialide structure, and sterigmata form), and molecular analysis utilizing 28 S-rRNA. The molecular evolutionary genetics analysis version 7.0 software (MEGA 7.0) was employed to align sequences from NCBI GenBank data to ascertain the taxonomic position of strains and construct the phylogenetic tree. The nucleotide sequences of fungal strains were submitted to GenBank to acquire their entry numbers. On the other hand, various cultural conditions like culture media type, time course of fermentation, initial medium pH, and carbon and nitrogen sources were optimized by one factor at a time for the biosurfactant production by *A. carneus* and *A. niger*.

### Emulsification index and oil spreading

To evaluate the emulsification index (E_24_), a 4 ml aliquot of the cell-free culture filtrate was mixed with 6 ml of toluene in a test tube. The mixture was agitated strongly for 2 min utilizing a tube shaker vortex. The ratio of emulsified toluene was assessed relative to the total volume after 24 h. The emulsification index of a 1% (w/v) sodium dodecyl sulfate (SDS) solution was also assessed for comparative analysis. E_24_ was calculated utilizing the formula^17^.

The oil spreading test was performed according to the procedures described previously^17,18^, with minor modifications. Fifty milliliters of sterilized distilled water were initially dispensed into a 9 cm glass Petri dish. A thin layer of sunflower seed oil (2 ml) was thereafter uniformly placed on the water’s surface. Subsequently, 100 µl of the cell-free supernatant containing the biosurfactant was carefully introduced to the center of the Petri dish. The area of oil displacement was subsequently measured.

### Biosurfactant extraction and characterization

For the extraction of biosurfactants, each flask was supplied with 100 ml of sterilized distilled water and subjected to agitation at 150 rpm for 1 h at 29 °C^19^. The suspension was centrifuged at 4 ºC at 5,000 rpm for 15 min. After centrifugation, the pellet underwent treatment with a 10 ml mixture of chloroform and methanol (2:1 volume ratio) and was then incubated at 30 °C in a shaking incubator at 200 rpm for 20 min. Following a 30min centrifugation at 5,000 rpm and 4 °C, the supernatant was let to evaporate through air drying^20^.

To do the phenol-H_2_SO_4_ test, 1 ml of cell-free supernatant was mixed with 1 ml of 5% phenol. Then, 3–5 ml of concentrated H_2_SO_4_ was added dropwise until an orange color appeared, which showed that the glycolipids were present in the crude biosurfactant^21^. For the Biuret test, a volume of two ml of cell-free supernatant was heated to 70 ºC and mixed with 1 M NaOH solution, and then CuSO_4_ solution was added to it drop-wise. Formation of a violet or pink color ring indicates the presence of lipopeptides^22^. Additionally, a phosphate test was carried out by mixing two ml of cell-free supernatant with 6–10 drops of 5 M HNO_3_ and heated to 70 °C, then 5% ammonium molybdate was added dropwise until the appearance of a yellow-colored precipitate, which indicates the phospholipid incidence^23^.

The biosurfactant composition was analyzed by comparing it to a methylated fatty acid ester mix standard using gas chromatography-mass spectrometry (GC-MS) with a QP2010 Ultra model detector. Each component was individually recognized according to its relative retention indices and cross-referenced with the Wiley Registry of Mass Spectral Data^24^. The biosurfactant was also characterized by FTIR analysis^25^. The column-purified biosurfactant was examined with a TENSOZ 27 (series no. 2887), covering the range of 500 to 4000 cm^− 1^, to identify the functional groups and bond types^26^.

### Polyaromatic hydrocarbons solubilization

A polyaromatic hydrocarbons **(**PAHs) solubilization assay was performed following the previously described methods^27,28^. Glass test tubes containing stock solutions of PAHs (anthracene, fluoranthene, fluorine, and pyrene) were filled with 60 µg of PAH each, and then the tubes were maintained open on a chemical fume hood to eliminate the solvent. The biosurfactant being tested was combined with 3.0 ml of assay buffer (comprising 20 mM Tris-HCl, pH 7.0) in concentrations ranging from 0 to 50 mg/ml. Assay buffer with solitary biosurfactant was utilized as a control. The tubes were sealed and left in a dark, 30 °C incubator with 200 rpm shaking. Filters measuring 1.2 μm were used to filter the samples (Whatman, No. 1). This filtrate was extracted in 2.0 ml using an equivalent volume of hexane. The aqueous solution and hexane phases of this emulsion were separated by centrifuging it for 10 min at 8,500 rpm. PAH concentration was measured spectrophotometrically at specific wavelengths of each compound: 350, 360, 239, and 263 nm wavelengths for anthracene, fluoranthene, pyrene, and fluorine, respectively. Hexane was used to extract the assay buffer, which included biosurfactant but no PAH, serving as a blank^28^.

### Applications of fungal biosurfactant

#### Antimicrobial activity

The antimicrobial activity of the biosurfactant extracted from fungi was achieved by using the agar well diffusion method^29,30^. Previously extracted compounds were examined against various actively growing standard target reference strains: *Bacillus subtilis* ATCC6633, *Staphylococcus aureus* ATCC25923, *Enterococcus faecalis* ATCC29212, *Escherichia coli* ATCC8739, *Salmonella typhi* ATCC6539, *K. pneumoniae* ATCC13883, *Candida albicans* ATCC10221, *Candida tropicalis* ATCC 750, and *Geotrichum candidum.* The crude biosurfactant was dissolved in distilled sterilized water to give 30 mg/ml in concentration. To generate a microbial lawn, a volume of 100 µl overnight culture of the test microbial isolates grown in Luria-Bertani broth was plated onto the previously described solid media. Then, 50 µl of the biosurfactant (30 mg/ml) was pipetted directly into the well. The antimicrobial tests were carried out with a positive control of two different antibiotics. Positive control was gentamicin (30 mg/ml) for bacteria and fluconazole (30 mg/ml) for fungi. All microbial strain tests were carried out in triplicate. All the plates were then incubated at 37 °C for 24–48 h. The diameter of the inhibition zone around the inoculated well was measured in mm^30^.

A half-maximal inhibitory concentration (IC_50_) test has been implemented. To complete 100 ml of nutrient broth in 250 ml Erlenmeyer flasks, we added varying concentrations of *A. carneus* or *A. niger* crude biosurfactants (30 mg/ml). The flasks were incubated at 35 °C for 48 h after being inoculated with 10 µl (3 × 10^5^ CFU/ml) of *B. subtilis* ATCC 6633. The bacterial pellets were extracted by centrifugation at 5000 rpm and dried at 80 °C for 2 h, following the measurement of the optical density at 600 nm.

#### Estimation of critical micelle concentration

The biosurfactants from *A. carneus* and *A. niger* cultures that were growing in the best conditions possible before show great ability to lower surface tension. A Du Nouy ring type tensiometer was used to measure the surface tension of diluted biosurfactants in various doses (2–120 mg/l)^31^. Pure water surface tension was measured as a control.

#### Elimination of lubricating oil from contaminated soils

The capacity of biosurfactants to extract oil from soil particles was assessed using 800 g of soil (1–2 mm in diameter) that had been acid-washed and combined with 50 ml of used lubricating oil. Samples comprising 20 g of contaminated soil were placed into 250 ml flasks and underwent the following treatments: 60 ml of distilled water (control), 60 ml of an aqueous solution of the biosurfactant Tween 80, and SDS. The studies were performed at the critical micelle concentration (CMC) of each crude biosurfactant, as well as at the CMC of each chemical, determined to be 18 mg/ml for SDS and 2 mg/ml for Tween 80, respectively. The samples were centrifuged for 20 min at 5,000 rpm to separate the dirt particles from the washing solution, after a 24-h incubation at 30 °C on a rotary shaker set to 200 rpm. Gravimetric analysis was employed to determine the quantity of material extracted from the soil using hexane. This enabled us to quantify the oil content in the soil particles subsequent to exposure to the biosurfactant^32^. The experiment was performed at different temperatures to assess the effect of temperature on oil recovery enhanced by biosurfactants.

### Statistical analysis

SPSS software (version 16 for Windows) was used for all statistical analyses. The obtained date was analyzed using one way ANOVA test. Finally, the means were compared using the Student–Newman–Keuls multiple range test at *p* ≤ 0.05.

## Results and discussion

### Screening for best biosurfactant producer fungal strains

Microorganisms synthesize a class of active compounds, which exhibit structural diversity^33–35^. Various industries, such as oil recovery, pollution bioremediation, healthcare, and food processing, have delineated their applications and prospective commercial uses^1^. Microbial surfactants demonstrate significant specificity, as evidenced by numerous studies on biosurfactant applications across various industries in the past decade, particularly in environmental remediation, including the extraction of heavy metals and organic compounds, as well as the bioremediation of hydrophobic substances^1,4^.

The results of this study revealed the existence of many physiologically active fungus sources in oil-contaminated soil samples. A total of eighty-nine morphologically different fungi were recovered from crude oil-contaminated soil and water samples in Kafr El-Sheikh governorate, Egypt, using PDA as a pure culture medium. The microbial isolation was successfully conducted utilizing potato dextrose broth culture medium augmented with 10 ml/l of sunflower seed oil. The emulsification index and oil spreading assay were employed to assess the capability of fungi to produce biosurfactants. Three species of *Aspergillus* had the highest efficacy in biosurfactant synthesis. The most promising fungi for biosurfactant synthesis were identified as *A. carneus* and *A. niger*. *Aspergillus* is among the most prevalent hydrocarbon degraders within fungal genera^36,37^. *Aspergillus* spp. has been widely employed to synthesize biosurfactants from several synthetic and biobased substrates^17^. The production of biosurfactants from the sea sponge *A. ustus* has been also reported^38^.

**Fig. 1:**
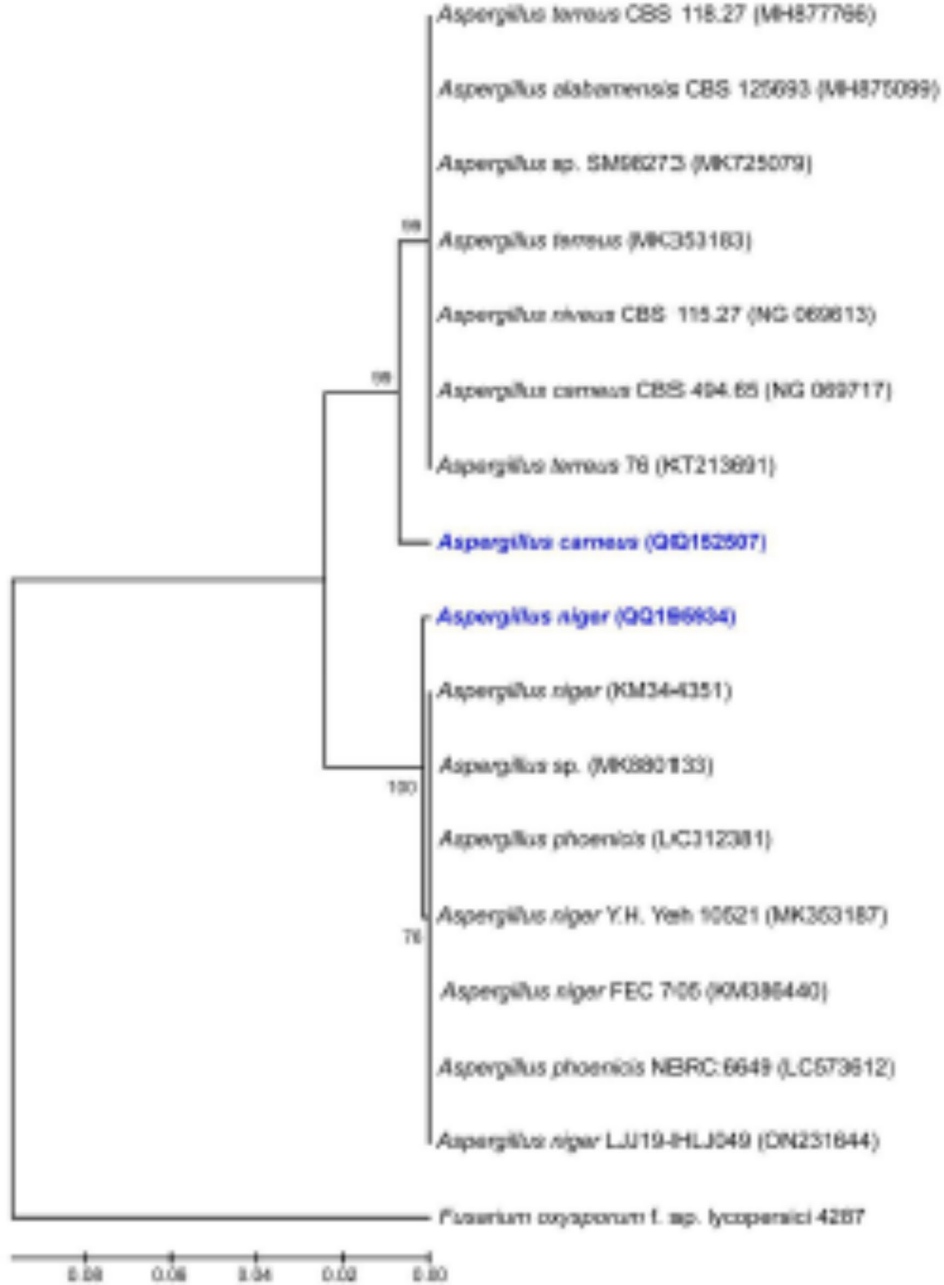
Phylogenetic analysis of the selected fungal isolates based on the results of PCR amplification of the 28 S-rRNA gene.

As for the phenotypic traits of the most potent isolates, *A. carneus* colonies are white at first but turn buff (brownish) over time, have a diameter of 1.2 cm, and have droplets that range in color from pale yellow to pale brown that come out of the cells. Microscopically, the conidial head is composed of large globose biserrate, brown conidia, thick-walled hyphae, and a smooth, brownish conidiophore that carries a globose vesicle. *A. niger* undergoes a notable transformation during its growth cycle. Initially, it appears as white colonies, but over time, it transitions into a distinct black color due to the production of conidial spores. Microscopic examination of *A. niger* reveals characteristic features that aid in its identification. *A. niger* conidia are smooth in texture and exhibit a range of colors. These conidia have radial heads and align in two rows, forming a biseriate pattern. This arrangement is a distinguishing feature of *A. niger* under microscopic observation. The *A. niger* conidiophores are also hyaline, which means they are clear or glass-like, and septate, which means they have cross-walls between the hyphae. The 28 S-rRNA genes of the selected fungi, *A. carneus* and *A. niger*, have been identified and entered into GenBank with accession numbers OQ152507 and OQ195934, respectively. After multiple sequence alignment between the obtained sequences, the result shown in Fig. 1 indicated that the tested sequences had 100% similarity PCR sequences with their respective species, *A. carneus* and *A. niger*.

### Optimization of biosurfactant production

#### Effect of culture media

Important characteristics of the culture medium should include strong selectivity towards the intended products over the unwanted ones. The availability of nutrient supplies for microorganisms and other environmental conditions has a significant impact on the production of biosurfactant^39^. The required data has shown that *A. carneus* OQ152507 and *A. niger* OQ195934 have a potential ability for bio-surfactant production as analyzed using common screening tests, such as E_24_ percentage, oil spreading, and surface tension tests^17,40^. The cultivation of *A. carneus* and *A. niger* revealed that these isolates possess the capability to efficiently produce biosurfactants under submerged culture conditions. The type of culture medium used significantly influenced both the emulsification index and the oil spreading test of *A. carneus* and *A. niger* biosurfactants (Table 1). *A. niger* surfactant, using starch nitrate medium, obtained the highest value (62.7%) for the emulsification index. *A. carneus* favored the glucose peptone medium for biosurfactant production, achieving an emulsification index of 57.27%. The oil spreading test conferred that the starch nitrate and glucose peptone media were the best for *A. carneus* and *A. niger*, respectively, to produce their biosurfactant. *A. carneus* showed the highest oil displacement (3.47 cm) when using the glucose peptone medium. *A. niger* biosurfactant’s oil displacement varied insignificantly, maintaining a mean of 3.30 cm. *Candida lipolytica* UCP0988 was capable of producing glycolipids in a medium of 5% animal fat and 2.5% corn steep liquor^41^. This resulted in the most significant reduction in surface tension, decreasing from 50 mN/m to 28 mN/m. Patil et al.^42^ employed 2% fresh coconut oil in an optimized mineral salt medium to synthesize biosurfactants from *Pseudomonas aeruginosa* F23, achieving a decrease in the surface tension of the culture medium from 45 mN/m to 31 mN/m. Substantial biosurfactant synthesis has been demonstrated by *Trichoderma citrinoviride* C1, HL, and B3 when cultured in Saunders medium and mineral-glucose-peptone medium^4^. They additionally demonstrated that a Saunders medium enriched with yeast biomass was more efficacious.

**Table 1:** Effect of different culture media on the fungal biosurfactant production.

Culture media	Emulsification index (%)		Oil spreading test (cm)	
	*A. carneus*	*A. niger*	*A. carneus*	*A. niger*
Glucose liquid	44.79^b^	32.50^d^	3.20^b^	2.10^c^
Glucose peptone broth	57.27^a^	50.40^b^	3.47^a^	2.50^b^
Yeast-malt extract broth	40.19^c^	45.53^c^	2.00^d^	2.13^c^
Bennett´s	41.26^c^	52.26^b^	2.00^d^	3.30^a^
Starch nitrate	42.11^c^	62.7^a^	2.53^c^	3.30^a^
Potato dextrose	42.17^c^	31.2^d^	2.00^d^	1.92^c^

Means with different letters in the same column differ significantly at *p* ≤0.05.

#### Effect of hydrocarbon oil

To study the effect of different hydrocarbon oils on the biosurfactant activity, the identified isolates, *A. carneus* and *A. niger*, were cultivated separately in an optimal free carbon source media (glucose peptone and starch nitrate) supplemented with 10 ml/l of certain hydrocarbon oils. All of the tested fungi’s supernatant showed emulsification activity, with an emulsification index ranging from 22.22 to 65.42% and an oil displacement ranging from 0.6 to 4.27 cm (Table 2). The emulsification index for each fungus varied depending on the hydrocarbon oil used. Kerosene had the highest emulsification index of *A. carneus*(65.42%), followed by petrol 80 (62.33%) for *A. niger*. According to the oil spreading test, kerosene was the most favorable substrate for *A. carneus* biosurfactant activity (4.27 cm), while *A. niger* had the highest oil spreading (4.13 cm) when using petrol 80 as a substrate. In conclusion, the presence of kerosene in the culture medium induced the highest emulsification index of *A. carneus* surfactant, followed by petrol 80 for *A. niger* surfactant. The addition of plant oils such as soybean, peanut, and olive oil to the culture medium led to the highest biosurfactant yields from *Pseudomonas*^43^. *Pseudomonas aeruginosa* achieved an emulsification index ranging from 55 to 60% against diesel when sunflower oil served as the carbon source^44^. Also, *B. subtilis* strain Al-Dhabi-130 made a crude biosurfactant that could remove 89% of the crude oil (which contained kerosene) from soil samples^45^. Wu et al.^46^ conducted a study that corroborated these findings, demonstrating that the *B. subtilis* SL biosurfactant exhibited a notable emulsification index of 67% with kerosene.

**Table 2:** Effect of different hydrocarbon oils on the fungal biosurfactant production.

Hydrocarbon oils	Emulsification index (%)		Oil spreading test (cm)	
	*A. carneus*	*A. niger*	*A. carneus*	*A. niger*
Sun flower seed	53.33^c^	42.33^c^	2.00^d^	2.60^d^
Paraffin	55.67^bc^	31.67^d^	2.60^c^	1.50^e^
Olive	29.18^d^	45.45^c^	1.93^d^	3.00^c^
Diesel	59.06^b^	28.00^d^	3.33^b^	1.00^f^
Petrol 80	24.36^d^	62.33^a^	1.50^e^	4.13^a^
Kerosene	65.42^a^	57.33^b^	4.27^a^	3.70^b^
Soy bean	24.33^d^	22.22^e^	1.00^f^	0.60^g^

Means with different letters in the same column differ significantly at *p* ≤0.05.

#### Effect of incubation period

**Fig. 2:**
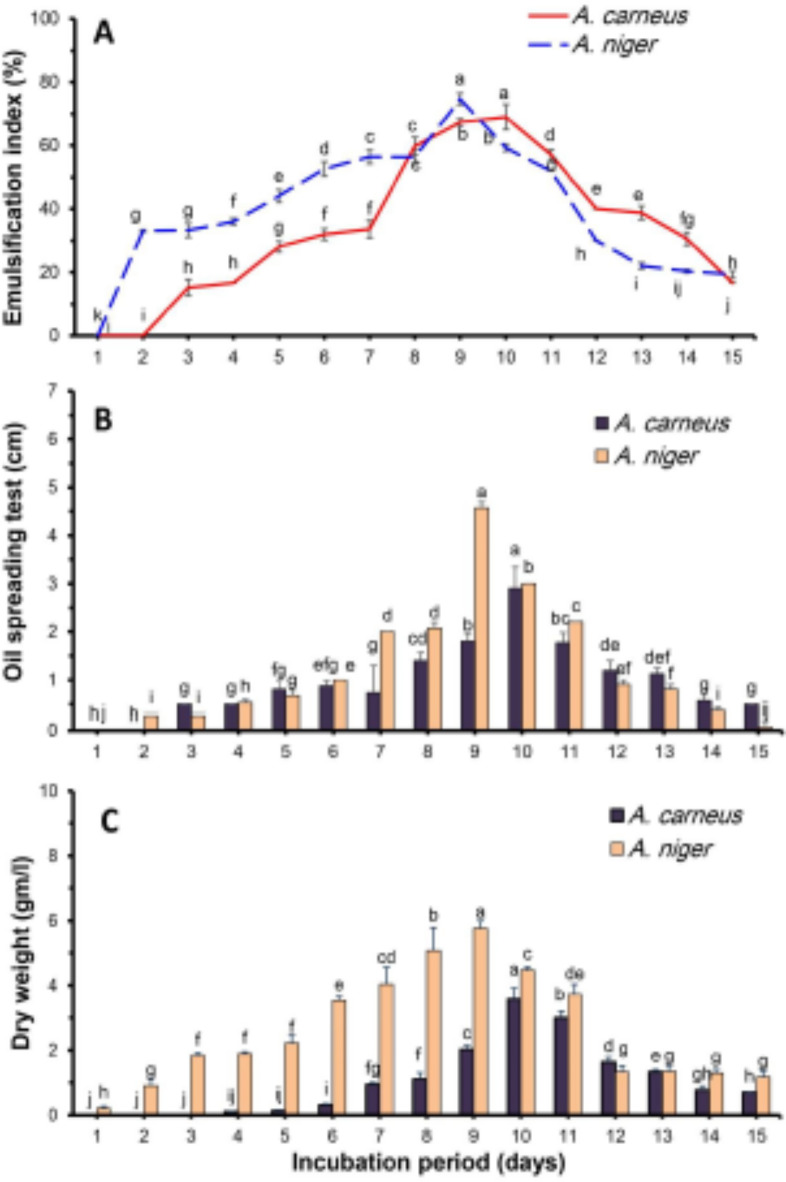
Effect of incubation period on emulsification index (**A**), oil spreading (**B**), and fungal biomass (**C**) of *A. carneus* and *A. niger* in submerged culture filtrate.

It became clear that as the incubation period increases, the biosurfactant potential expressed by emulsification and oil spreading increases to some extent, reaching the optimum period. Any increase in time leads to a reduction in activity. The highest emulsification index was obtained at 10 and 9 days of incubation for *A. carneus* and *A. niger*, respectively. They were 69, 74.70, and 68.33%, respectively (Fig. 2a). The oil displacement of the tested fungi had relatively the same pattern as the emulsification index, with the maximum value at 10 and 9 days for *A. carneus* and *A. niger*, respectively (Fig. 2b). In terms of microbial growth, the optimum dry weight was 3.57 g for *A. carneus* at the 10th day and 5.77 g for *A. niger* at the 9th day (Fig. 2c). Based on an increase in E_24_ and oil spreading of the culture filtrate, our data showed that the fungal strains began to excrete their biosurfactant following the lag phase. While the maximum biosurfactant activity was observed in the stationary phase from 9 to 11 days for *A. carneus* and from 8 to 11 days for *A. niger*. A lot of research has shown that some types of fungi, like *A. flavus*^47^, *A. ustus*^38^, *A. versicolor*^48^, *A. niger*^47^, and *Piper hispidum*^49^, produce biosurfactants during both their stationary phase and logarithmic phase. More precisely, *Penicillium* sp. 8CC2 reached its maximum biosurfactant production on the ninth day of development^50^. Similarly, the production of biosurfactant by *F. fujikuroi* UFSM-BAS-01, *Virgibacillus salarius* and *Bacillus licheniformis* has been documented^51–53^. Several researchers have also reported high biosurfactant production by yeasts during their stationary phase^54^. The production of *Bacillus subtilis* ANR 88 biosurfactant did not appear to be growth-associated^12^.

#### Effect of temperature and initial pH

Current data showed that the emulsification index, oil spreading test, and biomass of the tested fungi varied significantly with the temperature. Growing *A. carneus* and *A. niger* at 35 °C resulted in the highest emulsification index (E_24_ = 77.78% for *A. carneus* and 71.78% for *A. niger*). Additionally, the spread of oil varied depending on the temperature. Both *A. carneus* and *A. niger* yielded the highest value at 35 °C (Table 3). At 40 °C and 35 °C, *A. carneus* and *A. niger*, respectively, displayed the highest biomass. In conclusion, it was observed that the growth at 35 °C and 30 °C was favorable for *A. carneus* and *A. niger* to give the maximal productivity of biosurfactant and biomass. Several studies have demonstrated the positive influence of temperatures on the production of biosurfactants by bacteria and fungi^40^. For example, trehalose lipids with a lower surface tension (20.08 mN/m) are produced by *Fusarium fujikuroi* at 47 °C^55^ However, psychotolerant yeasts produce biosurfactants at 20 °C^56^. At submerged fermentation temperatures of 35 °C, the results show that the biosurfactant with the highest E_24_ and oil spreading was made. *Aspergillus* strains produced the maximum biosurfactant at 35 °C^17^. High moisture and moderate temperatures favor fungal growth. For example, field studies have demonstrated that fungi grow best in environments with high moisture and moderate temperatures. Temperature directly affects fungal growth and protein dynamics, which ultimately impact crop performance^57^. The optimum temperature for fungal growth varies among species, with some species growing best at temperatures around 25 °C, while others may require conditions up to 35 °C^58^. Other fungal species report that the ideal temperature for biosurfactant production is 30 °C^59^. Therefore, temperature plays a crucial role in both biosurfactant activity and fungal growth. Specific temperatures generally favor biosurfactant production, while moisture and temperature influence fungal growth, with moderate temperatures typically being the most favorable.

**Table 3:** Effect of temperature on biosurfactant activity and growth of *A. carneus* and *A. niger.*

Temperature (°C)	Emulsification index (%)		Oil spreading test (cm)		Dry weight (gm/l)	
	*A. carneus*	*A. niger*	*A. carneus*	*A. niger*	*A. carneus*	*A. niger*
15	0.00^g^	16.67^f^	0.00^e^	0.00^e^	0.09^e^	1.90^e^
20	16.33^f^	23.23^e^	1.00^d^	0.43^d^	0.53^d^	2.03^e^
25	32.78^e^	42.22^c^	0.20^e^	0.76^d^	1.27^cd^	2.99^c^
30	50.00^c^	61.44^b^	1.33^c^	3.80^b^	2.06^b^	4.09^b^
35	77.78^a^	71.78^a^	5.40^a^	4.33^a^	4.48^a^	5.21^a^
40	62.89^b^	38.18^d^	2.83^b^	1.67^c^	4.96^a^	2.29^de^
45	37.89^d^	21.42^e^	1.00^d^	0.50^d^	1.77^b^	2.5^cd^

Means with different letters in the same column differ significantly at *p* ≤0.05.

In submerged culture media, the initial pH values showed a significant effect on the biosurfactant activity and biomass of *A. carneus* and *A. niger*. All the variables (emulsification index, oil spreading test, and biomass) varied significantly according to the pH values (Table 4). *A. carneus* and *A. niger* recorded the highest and best emulsification activity at pH 7. It was 61.11% and 69.0% for *A. carneus* and *A. niger*, respectively. The results indicated that the optimum pH was 7 for the emulsification activity, oil spreading, and biomass productivity for *A. carneus* and* A.niger*(Table 4). The obtained data aligned with the findings of Alyousif et al.^60^, who observed that *Pseudomonas* sp. and* B.subtilis* produced their maximum biosurfactant at pH 7. Changing in the pH to acidity or alkaline regions decreased the biosurfactant activity of *A. carneus* and *A. nige.* These results almost align with previous findings by Saimmai et al.^61^, which confirmed a decrease in the emulsification activity of *Oleomonas sagaranensis* AT18 surfactant with increasing acidity. Fouda et al.^62^ reported that raising the pH from 8 to 10 resulted in lower biosurfactant productivity in *P. aeruginosa* and *B. cereus*.

**Table 4:** Effect of initial pH on biosurfactant activity and growth of *A. carneus* and *A. niger*.

pH	Emulsification index (%)		Oil spreading test (cm)		Dry weight (gm/l)	
	*A. carneus*	*A. niger*	*A. carneus*	*A. niger*	*A. carneus*	*A. niger*
4	35.85^c^	30.00^e^	1.30^c^	0.50^e^	4.24^bc^	1.81^d^
5	37.28^c^	36.33^d^	1.50^c^	0.70^e^	4.15^bc^	2.82^c^
6	54.20^b^	64.12^b^	1.83^b^	4.57^b^	4.96^b^	7.54^a^
7	61.11^a^	69.00^a^	4.47^a^	5.37^a^	6.52^a^	8.08^a^
8	53.00^b^	45.33^c^	1.80^b^	3.13^c^	3.36^cd^	7.64^a^
9	28.67^d^	43.52^c^	0.50^d^	1.83^d^	1.93^d^	5.62^b^

Means with different letters in the same column differ significantly at *p* ≤0.05.

#### Effect of carbon and nitrogen sources

**Fig. 3:**
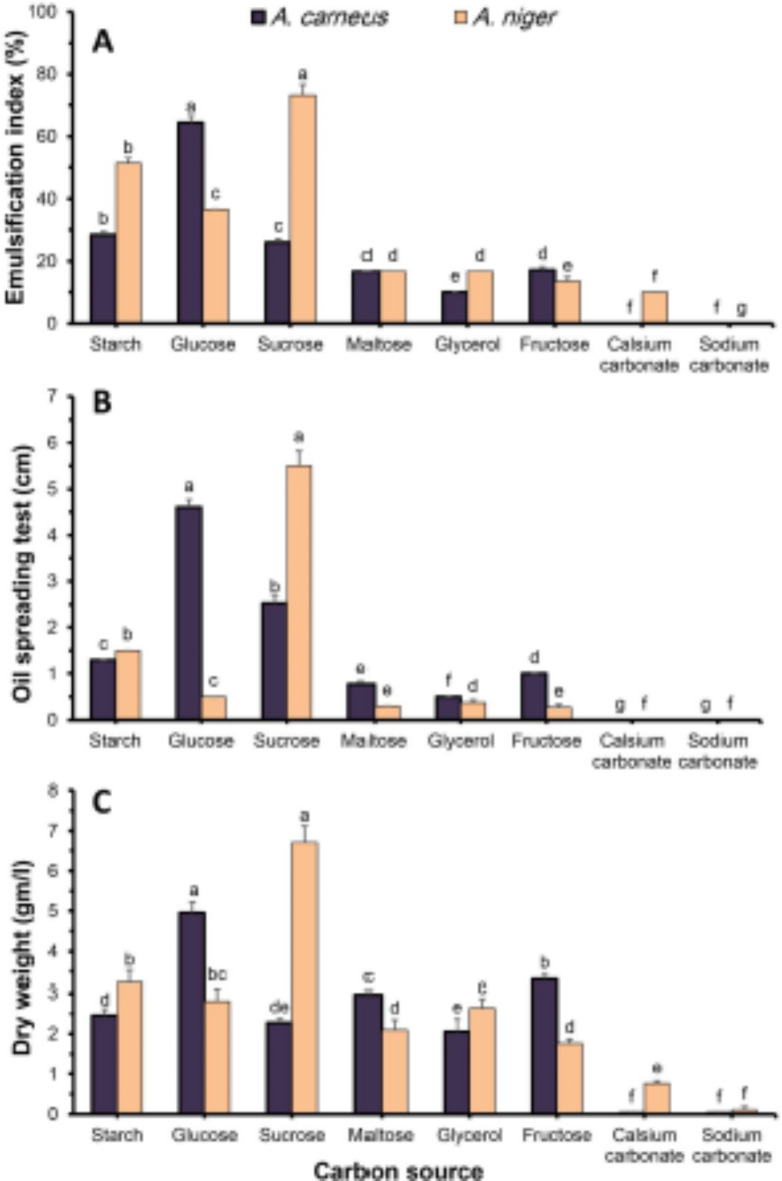
Effect of different carbon sources on emulsification index (**A**), oil spreading (**B**), and fungal biomass (**C**) of *A. carneus* and *A. nige*r in submerged culture filtrate.

The carbon consumption rate of microorganisms during cultivation often regulates growth and secondary biochemical production, including biosurfactants^63^. Microorganisms can produce biosurfactants in aqueous media by adding carbon sources such as glucose, fructose, glycerol, mannitol, different oils, and feedstock wastes. Different renewable feedstocks as substrates in fermentation bioprocesses have been studied. Most of the carbon used to make biosurfactants comes from carbohydrates, such as simple sugar, starch, and plant sugar-based carbohydrates^64,65^. Various biosurfactant producers can benefit from using pure carbohydrates as carbon sources, such as glucose, sucrose, and glycerol^66^. Data shown in Fig. 3 indicated that the organic carbon sources were much better than inorganic ones. To make the most biosurfactant activity (E_24_ = 64.39%; Fig. 3a), oil displacement (4.60 cm; Fig. 3b); and biomass production (4.95 g/ml; Fig. 3c), *A. carneus* used glucose optimally. *P. aeruginosa* MTCC 7815 can use glucose better than other carbon sources to make an emulsification index of 76.77% and a surface tension of 34.53 mN/m^65^. These results support their findings. On the other hand, sucrose was the best carbon source for *A. niger* growth and biosurfactant productivity, with an emulsification index of 73.33% (Fig. 3a), oil spreading of 5.5 cm (Fig. 3b), and biomass of 6.70 g/ml (Fig. 3c). In the same context, *P. aeruginosa* UMTKB-5 produced high amounts of rhamnolipid (above 250 mg/l) when grown in either glucose or sucrose medium^67^.

**Fig. 4:**
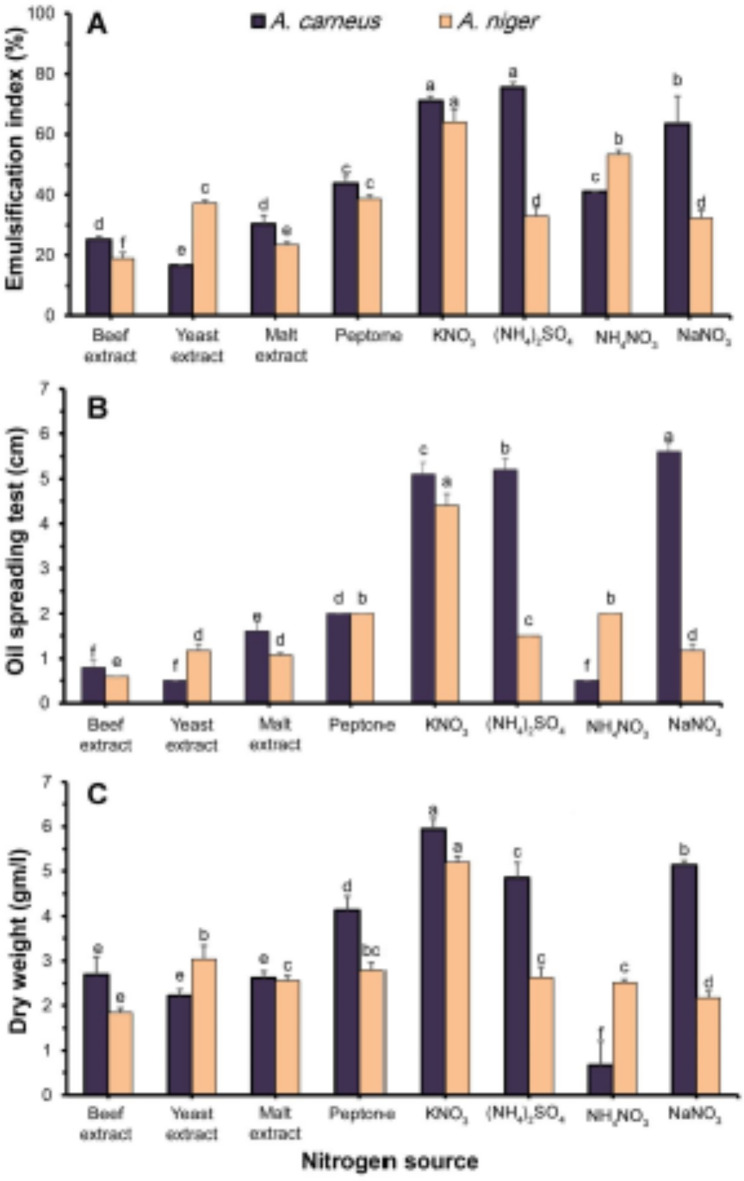
Effect of different nitrogen sources on emulsification index (**A**), oil spreading (**B**), and fungal biomass (**C**) of *A. carneus* and *A. niger* in submerged culture filtrate.

Several inorganic and organic nitrogen sources can influence biosurfactant production. Commonly utilized inorganic sources include nitrate and ammonium salts, while organic sources include corn-steep liquor, yeast extract, and urea^13^. In this study, eight nitrogen compounds were used to study their effect on biosurfactant activity and biomass production of the tested fungi (Fig. 4). Ammonium sulfate and potassium nitrate were the best nitrogen sources for the emulsification index of *A. carneus* and *A. niger*, respectively (Fig. 4a). For *A. carneus* and *A. niger*, it was 75.54 and 64.00%, respectively. These results are similar to those found by Adamczak and Bednarski^68^, who found that ammonium salts are the best nitrogen sources for *Candida antartica* to make biosurfactants. Additionally, Banat et al.^69^ demonstrated that ammonium sulfate was similarly the most effective nitrogen source for biosurfactant production from *Serratia marcescens*. However, potassium nitrate yielded the highest biosurfactant activity for *A. niger*. Alyousif et al.^60^ identified NaNO_3_ as the ideal nitrogen source for *P. aeruginosa* to produce biosurfactants. Some *B. subtilis* strains were unable to produce biosurfactants using (NH_4_)_2_SO_4_ or KNO_3_, but they could use NaNO_3_, NH_4_NO_3_, or KNO_3_^70,71^. Ma et al.^72^ validated that inorganic nitrogen sources were more suitable for sophorolipid production than organic sources. Peptone also reduced the emulsification index of *Aspergillus favus* biosurfactants^73^. The optimized *A. carneus* OQ152507 and *A. niger* OQ195934 biosurfactants resulted in a significant decrease in water surface tension. These results were in accordance with the data obtained from biosurfactant of *Fusarium fujikuroi*, *Leucobacter komagatae*, and mutated strain of *B. subtilis*^17,28^. While, among the three nitrogen sources, *Lysinibacillus fusiformis* biosurfactants had the highest emulsification index (78.31 ± 0.87%) using urea followed by yeast extract^58^.

#### Effect of sugar, nitrogen, and critical micelle concentrations

Glucose was the best carbon source for *A. carneus*, while sucrose was favored by *A. niger* to produce the highest biosurfactant activity and the fungal biomass. *A. carneus* recorded the highest emulsification index (61.07%) with a concentration of thirty grams of glucose, while *A. niger*’s supernatant demonstrated the highest emulsification index (64.33%) with a concentration of 30 g/l of sucrose. Concerning the oil spreading test, 30 and 35 g/l of the preferred sugar were the best carbon source concentrations, giving the highest oil displacement: 3.5 and 4.73 cm for *A. carneus* and *A. niger*, respectively. The highest biomass for *A. carneus* and *A. niger* was obtained using 35 g/l of the favorable carbon source for each microbial isolate (Table 5). For biosurfactant production, several microorganisms evaluated various raw materials containing different concentrations of carbon sources. For example, a medium containing pure cashew apple juice supplemented with 10 g/l glucose and 8.7 g/l fructose resulted in a twofold increase in *B. subtilis* LAMI005 biosurfactant production compared to a mineral medium containing sugar at a concentration of 12.05 g/l^74^. Roberta et al.^75^ achieved the best biosurfactant production of *Coprinus* sp. FS-4.1 using potato dextrose broth containing both 1% glucose and 2% glycerol. Using glucose (10%) as the sole carbon source for *Rhodotorula babjevae* YS3 growth increased the production of biocurfactants. This decreased the surface tension from 70 to 32.6 mN/m^50^. Adding glucose (2%) to the paneer whey medium increased the yield of *pseudomonas aeruginosa* SR17 rhamnolipids to 4.8 g/l^76^.

**Table 5:** Effect of different sugar concentrations on fungal biosurfactant activity and growth.

Sugar conc. (gm/l)	Emulsification index (%)		Oil spreading test (cm)		Dry weight (gm/l)	
	*A. carneus*	*A. niger*	*A. carneus*	*A. niger*	*A. carneus*	*A. niger*
5	14.45^e^	12.61^d^	0.63^e^	0.13^e^	1.43^e^	1.87^c^
10	18.36^d^	13.84^d^	0.7^e^	1.20^d^	2.93^d^	2.30^bc^
15	33.33^c^	34.89^c^	1.53^d^	2.53^c^	3.00^cd^	2.93^b^
20	57.90^a^	58.82^b^	3.07^c^	3.20^bc^	3.93^bc^	4.60^a^
25	59.63^a^	60.23^b^	3.17^bc^	3.40^b^	4.37^ab^	4.47^a^
30	61.07^a^	64.49^a^	3.50^a^	4.13^a^	4.73^ab^	4.83^a^
35	40.97^b^	67.73^a^	1.37^d^	4.73^a^	5.00^a^	5.10^a^
40	31.87^c^	38.70^c^	0.47^e^	1.77^d^	3.30^cd^	3.07^b^

Means with different letters in the same column differ significantly at *p* ≤0.05.

The activity of *A. carneus* and *A. niger* to produce their surfactant depending upon the different nitrogen source concentrations was evaluated. The emulsification index, oil spreading, and fungal biomass of the two fungi were used to express the biosurfactant activity, revealing significant variations between nitrogen concentrations ranging between 1 and 4 g/l. In this experiment, *A. carneus* preferred ammonium sulfate as a nitrogen source, while *A. niger* preferred potassium nitrate. A dramatic increase was observed in emulsification index and oil spreading with increasing the nitrogen concentration, with a peak at a concentration of 2.5 g/l for *A. carneus* and *A. niger*. The highest biomass was obtained for *A. carneus* and *A. niger* at 2.5 g of the favorable nitrogen source for each microbial fungus (Table 6). *Ustilago maydis* produced different concentrations of cellobiose and mannosylerythritol lipids under nitrogen starvation^77^. The biosurfactants from *A. carneus* and *A. niger* cultures that were growing in the best conditions possible before have great properties for lowering surface tension. The water tension surface (71.57 mN/m) decreased to 33.3 and 30.86 mN/m, respectively, when 120 mg/l of *A. carneus* and *A. niger* biosurfactant were used (Table 7). This indicates that the crude biosurfactant of *A. carneus* and *A. niger* reduced the surface tension by 53.5 and 56.9%, respectively. The critical micelle concentration of biosurfactant produced from *A. carneus* was 30 mg/l, which decreased the water surface tension to 36.7 mN/m, whereas in the case of *A. niger*, it was 40 mg/l, which decreased the water surface tension to 32.73 mN/m. On the other hand, *Bacillus megaterium* pL6 had a CMC of 100 mg/l^78^, while *Agrobacterium fabrum* SLAJ731 had a CMC of 650 mg/l^79^.

**Table 6:** Effect of different nitrogen concentrations on fungal biosurfactant activity and growth.

Nitrogen source concentration (gm/l)	Emulsification index (%)		Oil spreading test (cm)		Dry weight (gm/l)	
	*A. carneus*	*A. niger*	*A. carneus*	*A. niger*	*A. carneus*	*A. niger*
1	19.02^e^	26.15^e^	0.40^e^	0.50^f^	1.23^d^	2.20^d^
1.5	43.07^c^	34.92^d^	1.27^cd^	1.20^e^	2.63^bc^	3.00^c^
2	49.82^b^	40.23^c^	2.17^b^	1.40^de^	3.13^ab^	3.10^c^
2.5	58.70^a^	58.95^a^	3.33^a^	3.37^a^	3.67^a^	5.10^a^
3	49.30^b^	54.21^ab^	2.07^b^	2.60^bc^	2.93^b^	4.13^b^
3.5	41.33^c^	55.44^ab^	1.43^c^	2.70^b^	2.17^c^	3.13^c^
4	31.45^d^	52.58^b^	0.93^d^	1.97^cd^	1.97^c^	2.70^cd^

Means with different letters in the same column differ significantly at *p* ≤0.05.

**Table 7:** Critical micelle concentration of *A. carneus* and *A. niger* biosurfactant.

Biosurfactant concentration (mg/l)	Surface tension (mN/m)	
	*A. carneus*	*A. niger*
0	71.57^a^	71.57^a^
2	62.81^b^	50.02^b^
4	58.28^c^	46.25^c^
6	55.34^d^	43.79^d^
8	50.47^e^	40.48^e^
10	46.65^f^	37.53^f^
20	39.25^g^	34.04^g^
30	36.70^h^	33.24^h^
40	36.67^hi^	32.73^h^
50	36.62^hi^	32.10^i^
60	36.60^ij^	32.08^i^
70	36.53^j^	31.36^j^
80	35.50^k^	31.28^j^
90	35.37^l^	31.18^j^
100	34.74^m^	31.08^j^
110	33.41^n^	31.00^j^
120	33.33^n^	30.86^j^

Means with different letters in the same column differ significantly at *p* ≤0.05.

### Biosurfactant application

#### Antimicrobial activity

**Fig. 5:**
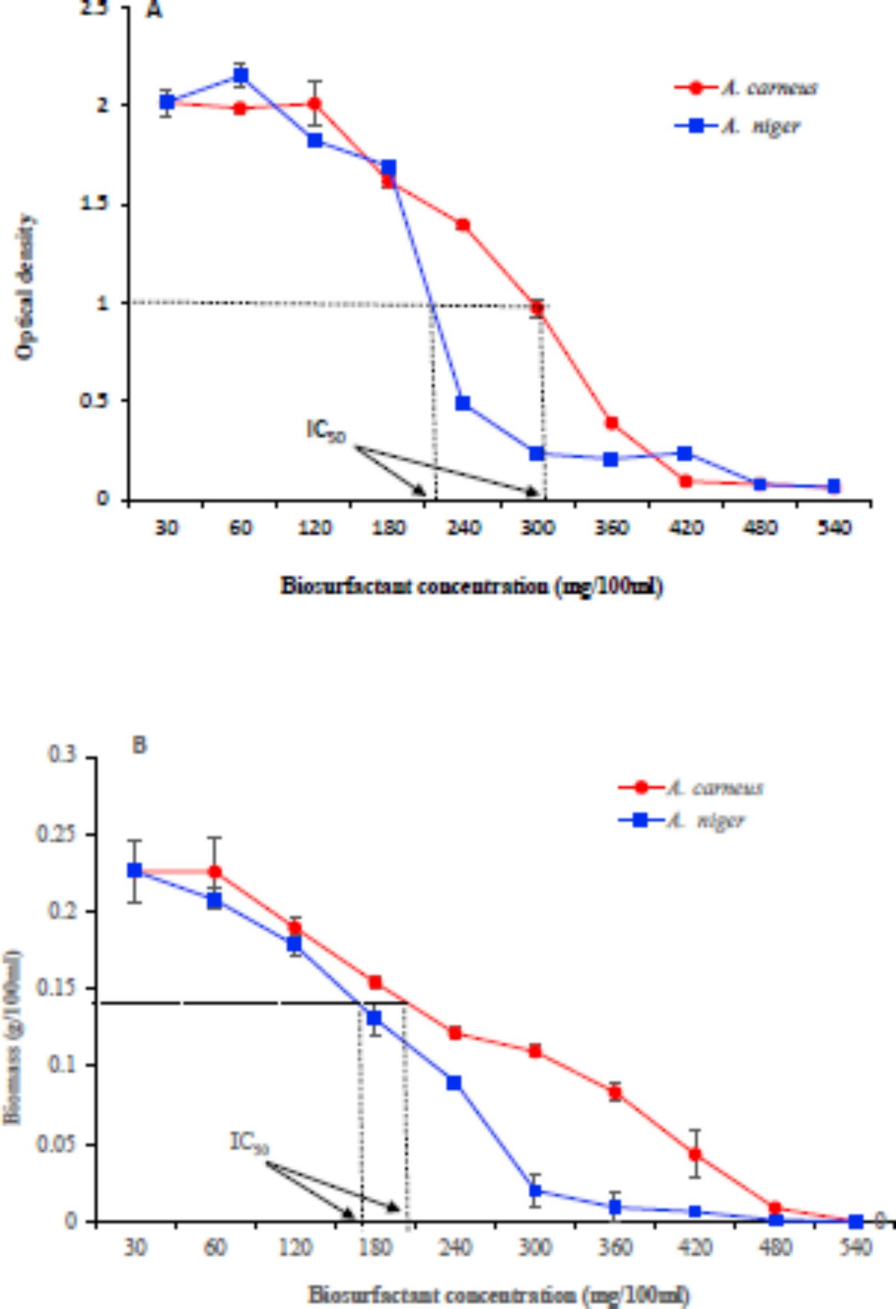
Effect of *A. carneus* and *A. niger* biosurfactant concentration on bacterial growth of *B. subtilis* ATCC 6633 optical density (**A**) and biomass (**B**).

The biosurfactant antimicrobial activity was carried out against nine selected pathogenic microbes (Table 8). The biosurfactant from *A. carneus* and *A. niger* demonstrated significant antimicrobial activity against all tested microbes. It varied significantly according to the tested microbes (Table 8). *A. niger* biosurfactant exhibited the highest inhibitory effect against *S. aureus* ATCCG538 (40 mm), followed by *B. subtilis* ATCC6633 (39.07 mm), *K. pneumoniae* ATCC13883 (35 mm), and *E. coli* ATCC8739 (33 mm), while *A. carneus* biosurfactant demonstrated the highest effect against *B. subtilis* ATCC6633 (34.03 mm) in comparison to the control. The effect of the extracted crude biosurfactant of *A. carneus* and *A. niger* on the growth of *(B) subtilis* ATCC 6633 was studied by determining its optical density and biomass. Regarding the optical density, the results indicated that *A. carneus* and *A. niger* biosurfactant caused a relatively complete growth inhibition at 420 and 300 mg/100 ml, respectively (Fig. 5A). The highest concentration of *(A) niger* biosurfactant that stopped the growth of *B. subtilis* ATCC 6633 was 300 mg/100 ml (Fig. 5B). Conversely, using *A. carneus* biosurfactant yielded results between 420 and 480 mg/100 mL (Fig. 5B). The IC_50_ was 300 mg/100 ml for *A. carneus* biosurfactant and 220 mg/100 ml for *A. niger* biosurfactant (Fig. 5A). The biomass measurement revealed that it was approximately 205 and 170 mg/100 ml, respectively (Fig. 5B). Many studies proved the antimicrobial activity of various biosurfactants. Serrawettin W1, a biosurfactant found in *Serratia marcescens*, can inhibit Gram-positive bacteria^80^. A lot of different types of bacteria, including *Shigella dysenteriae*, *P. aeruginosa*, *S. aureus*, and *Micrococcus luteus*, can be inhibited by Serrawettin W2, a biosurfactant found in *Serratia surfactantfaciens* YD25T^81^. Crude biosurfactant extracts from *Lactobacillus jensenii* and *Lactobacillus rhamnosus* could inhibit clinical multidrug-resistant strains of *S. aureus*, *E. coli* EC433, and *Acinetobacter baumannii*^82^. The broad antimicrobial action of sophorolipids of *Candida bombicola* ATCC 22,214 against several microorganisms, including *E. coli*, *P. aeruginosa*, and *S. aureus* has been reported^83^. Morais et al.^84^ demonstrated the biosurfactant using *L. jensenii* P6A and *L. gasseri* P65. Liu et al.^85^ demonstrated that the biosurfactant surfactin C15 effectively inhibited *C. albicans* SC5314. Another investigation found that biosurfactants made by *Lactobacillus* spp. had antibacterial effects on oral streptococci^86^.

**Table 8:** Antimicrobial activity of *A. carneus* and *A. niger* biosurfactant.

Microorganisms	Inhibition zone (mm)		
	*A. carneus*	*A. niger*	Control*
*B. subtilis* ATCC 6633	34.03^a^	39.07^b^	25.07^b^
*S. aureus* ATCC 6538	27.00^e^	40.00^a^	22.03^d^
*E. faecalis* ATCC 10,541	32.93^b^	31.03^e^	30.13^a^
*E. coli* ATCC 8739	30.97^d^	32.97^d^	22.93^c^
*S. typhi* ATCC 6539	24.03^c^	29.90^f^	25.17^b^
*K. pneumoniae* ATCC13883	32.03^c^	35.03^c^	19.13^g^
*C. albicans* ATCC 10,221	24.03^f^	27.90^g^	20.97^e^
*C. Tropicalis* ATCC 750	23.97^f^	30.00^f^	22.17^d^
*G. candidum*	23.10^g^	21.90^h^	19.90^f^

*Positive control was Gentamicin at 30 mg/ml for bacterial isolates and Fluconazole at 30 mg/ml for yeasts and filamentous fungi.

Means with different letters in the same column differ significantly at *p* ≤0.05.

#### Biosurfactant effect

Solubilization tests with biosurfactant concentrations between 5 and 30 mg/ml were done to evaluate the effect of the biosurfactant on the apparent water solubility of PAHs. An increase in biosurfactant concentration typically leads to a rise in the apparent solubility of PAHs. The incorporation of *A. carneus* or *A. niger* biosurfactant enhances the solubility of fluoranthene, pyrene, anthracene, and fluorine in aqueous solutions (Table 9). The maximum degree of solubilization was observed at a concentration of 30 mg/ml for both biosurfactants tested. Treatment with 30 mg/ml of *A. carneus* biosurfactant led to a substantial enhancement in the solubility of pyrene, fluoranthene, anthracene, and fluorine by over 17, 27, 26, and 3 times, respectively, in comparison to pure water. Conversely, a 27-fold enhancement in the solubility of PAHs for anthracene has been obtained when treated with *A. niger* biosurfactant, relative to its control (Table 9). Results have proven that the *A. carneus* or *A. niger* biosurfactant improved the solubility of PHAs, fluoranthene, pyrene, anthracene, and fluorine in water. The solubility of PHAs was directly proportional to the biosurfactant concentration. Aafter 16 days of incubation, *Phanerochaete sordida* YK-624 totally destroyed pyrene at the low concentration^87^. Biosurfactants from *A. carneus* and *A. niger* can break up hydrocarbons, which lowers the surface tension and makes it easier for oil to move from soil particles^88^. Naphthalene, phenanthrene, and pyrene became more soluble as the levels of the biosurfactant rhamnolipid biosurfactant in *Bacillus* Lz-2 increased^89^. The use of biosurfactants may increase PAH biodegradation efficiency more than chemical surfactants. for example, soil that was contaminated with PAHs and treated with rhamnolipid, a biosurfactant, had a biodegradation rate that was 95%, which was higher than the biodegradation rates of SDS (90%) and Tween 80 (92%)^90^. Also, a basic biosurfactant from *Bacillus stratosphericus*,* B. subtilis*, *B. megaterium*, and *P. aeruginosa* improved the breakdown of PAHs in soil that had been contaminated with creosote^91^. Zhou et al.^92^ observed a high degrading efficiency of an aqueous system containing pyrene, phenanthrene, benzo[b]fluoranthene, and benzo[a]pyrene in *Mycobacterium gilvum* MI, *Mycobacterium* sp. ZL7, and *Rhodococcus rhodochrous* Q3. In the same context, a number of fungi can breakdown PAHs. The white rot fungus *P. ostreatus* can break down pyrene, phenanthrene, fluorene, anthracene, and benzo[a]pyrene in water^93^. A combination of four PAHs (chrysene, phenanthrene, naphthalene, and benzo(a)pyrene) could be broken down by the *Fusarium solani* strain HESHAM-1 isolated from soil contaminated with oil^94^.

**Table 9:** Effect of different concentration of crude biosurfactant of *A. carneus* and *A. niger* on the solubility of polyaromatic hydrocarbons.

Biosurfactant conc. (mg/ml)	Solubility of PAHs* (mg/l)							
	*A. carneus*				*A. niger*			
	Fluoranthene	Pyrene	Anthracene	Fluorene	Fluoranthene	Pyrene	Anthracene	Fluorene
0	0.26^g^	0.17^f^	0.08^g^	2.01^g^	0.24^g^	0.17^g^	0.06^g^	1.76^g^
5	0.99^f^	0.89^e^	0.23^f^	2.13^f^	0.81^f^	1.53^f^	0.17^f^	2.08^f^
10	1.86^e^	1.08^e^	0.54^e^	3.15^e^	1.34^e^	1.86^e^	0.40^e^	2.90^e^
15	2.87^d^	1.41^d^	0.84^d^	3.91^d^	1.77^d^	2.34^d^	0.82^d^	3.69^d^
20	3.61^c^	2.24^c^	1.13^c^	4.63^c^	2.70^c^	2.70^c^	0.94^c^	4.54^c^
25	5.00^b^	2.59^b^	1.52^b^	5.35^b^	3.73^b^	2.91^b^	1.12^b^	5.80^b^
30	7.12^a^	2.96^a^	2.14^a^	6.92^a^	4.27^a^	3.08^a^	1.63^a^	7.09^a^

Means with different letters in the same column differ significantly at *p* ≤0.05.

#### Removal of lubricating oil from the contaminated soil

The ability of the tested biosurfactants of *A. carneus* and *A. niger* to remove lubricating oil from the contaminated soil was examined compared to certain synthetic surfactants, that is, a nonionic surfactant tween 80 and an ionic surfactant SDS. The recovery percentage of lubricating oil by both synthetic and biosurfactant varied significantly depending on the temperature. Table 10 illustrates the varying effectiveness of different surfactants in recovering oil from contaminated soil under different temperature conditions. Tween 80 demonstrated significant recovery rates, retrieving 22% of the oil at 25 °C, 38% at ambient temperature, 67% at 45 °C, and 75% at 60 °C. In contrast, the synthetic surfactant SDS showed lower efficacy, with recovery rates ranging from 7 to 12% across the temperature range. The control group that received distilled water showed minimal recovery, ranging from 10 to 27%. Additionally, *A. carneus* biosurfactant recovered 25% of the oil at 25 °C, 37% at ambient temperature, 64% at 45 °C, and 76% at 60 °C, while *A. niger* biosurfactant retrieved 32% of the oil at 25 °C, 40% at room temperature (30 °C), 63% at 45 °C, and 80% at 60 °C. Generally, biosurfactants offer a promising alternative for enhancing oil recovery due to their unique properties and environmental benefits. Two of the most popular forms of biosurfactants, rhamnolipid and cyclohexanone, have successfully removed oil molecules from soil^95^. People have widely used them to remediate oil pollutants in contaminated soil. These results showed that biosurfactants are better than synthetic surfactants like SDS and Tween at getting oil pollutants out of contaminated soil. In this work, we investigated biosurfactants that could serve as biostimulator agents for oil-contaminated soil bioremediation.

**Table 10:** Removal of lubricating oil from the contaminated soil.

Temperature (°C)	Chemical surfactant			Biosurfactant	
	Control	SDS	Tween 80	*A. carneus*	*A. niger*
25	0.90^a^	0.93^a^	0.78^a^	0.75^a^	0.68^a^
30	0.85^b^	0.91^a^	0.62^b^	0.63^b^	0.60^b^
45	0.78^c^	0.89^b^	0.43^c^	0.36^c^	0.37^c^
60	0.73^d^	0.88^b^	0.25^d^	0.24^d^	0.20^d^

Means with different letters in the same column differ significantly at *p* ≤0.05.

### Biosurfactant characterization

The chemical nature of a biosurfactant can be determined by the phenol-H_2_SO_4_ test to detect glycolipids, the biuret test to detect lipopeptides, and the phosphate test to detect phospholipids. First, it was found that the biosurfactants from *A. carneus* and *A. niger* are made up of glycolipid and lipopeptide molecules. The orange color showed that glycolipids were present in the biosurfactant crude of *A. carneus* and *A. niger* biosurfactant by phenol-H_2_SO_4_ test. Also, phospholipids were detected in the tested biosurfactant (Table 11). The negative results of the Biuret test indicated the absence of lipopeptides in *A. carneus* and *A. niger* biosurfactants.

**Table 11:** Detection of chemical compounds in *A. carneus* and *A. niger* biosurfactant.

Compound	Biosurfactant	
	*A. carneus*	*A. niger*
Glucolipids	+	+
Lipopeptides	−	−
Phospholipids	+	+

**Fig. 6:**
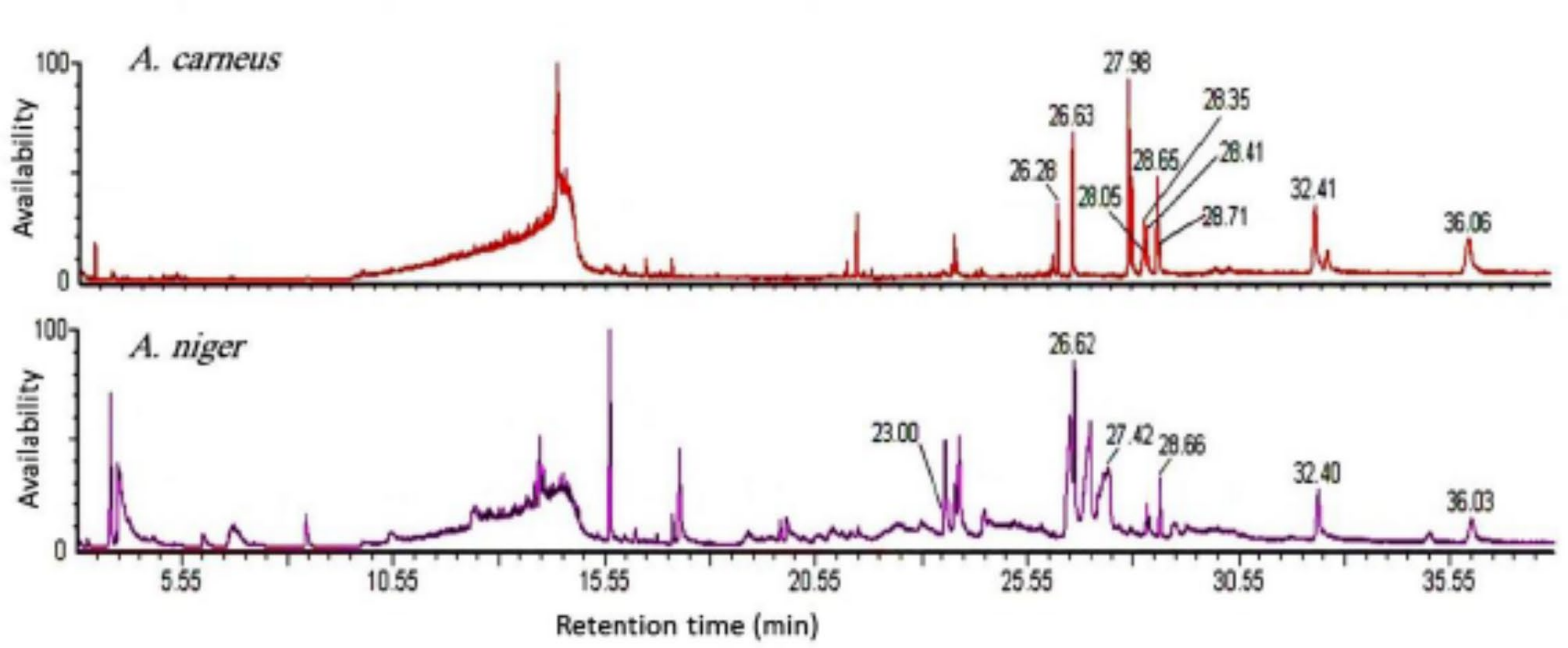
GC-MS analysis of *A. carneus* and *A. niger* culture filtrate.

A GC-MS analysis revealed the presence of 50 different chemical compounds. They have different retention times, ranging from 3.429 to 36.057 min. The highest percentage content (area %) of the compounds are as follows: DL-Ethionine (12.48%), 1,2,3-Propanetriol, monoacetate (12.382%), Glycerin (6.353%), Methanamine, N-methoxy (4.76%), Octadecanoic acid, 2,3-dihydroxypropyl ester (2.787%), Hexadecanoic acid, 2-hydroxy-1- 5(hydroxymethyl) ethyl ester (2.453%), 9,12-octadecadienoic acid, methyl ester, (E, E)- (2.143%), n-Hexadecanoic acid (1.942%), 9-Octadecenoic acid (Z)-, methyl ester (1.114%), and trans-13-octadecenoic acid (0.467%). Out of these compounds, the GC-MS analysis identified ten hydrophobic compounds listed in Table 12 with retention times ranging from 26.278 to 36.057 min (Fig. 6). These hydrophobic tails may represent a part of certain biosurfactant(s). In terms of area percentages, 9,12-Octadecadienoic acid, methyl ester, (E, E)- has the most content (2.923%). It is followed by Octadecanoic acid, 2,3-dihydroxypropyl ester (2.787%), Palmitic acid ME P1297 (2.774%), and Hexadecanoic acid, 2-hydroxy-1(hydroxymethyl)ethyl ester (2.453%). These results suggest that the sample contains a diverse range of chemical compounds, with varying retention times and percentage contents. The presence of hydrophobic compounds within the biosurfactant(s) indicates that they may have a significant impact on the overall properties and effectiveness of the biosurfactants. More research into these compounds and how they work can help us understand what biosurfactants are and how they might be used in different fields, like food, medicine, cleaning up the environment, and oil and gas.

**Table 12:** Hydrophobic tail compounds detected by GC-MS analysis.

Structure	Compound name	RT	Area %
*A. carneus*			
C_16__32_O_2_	Palmitic acid ME P1297	26.278 – 26.633	2.774
C_19_H_34_O_2_	9,12-Octadecadienoic acid, methyl ester, (E, E)-	27.984 – 28.354	2.923
C_19_H_36_O_2_	9-Octadecenoic acid (Z)-, methyl ester	28.054	1.114
C_18_H_34_O_2_	Trans-13-Octadecenoic acid	28.414	0.467
C_18_H_36_O_2_	Octadecanoic acid	28.654	1.635
C_20_H_38_O_2_	Ethyl Oleate	28.709	0.54
C_16_H_34_O	2-Hexadecanol	30.019	0.275
C_26_H_54_	Octadecane, 3-ethyl-5-(2-ethylbutyl)-	32.355	0.296
C_19_H_38_O_4_	Hexadecanoic acid, 2-hydroxy-1(hydroxymethyl)ethyl ester	32.405	2.453
C_21_H_42_O_4_	Octadecanoic acid, 2,3-dihydroxypropyl ester	36.057	2.787
*A. niger*			
C_18_H_34_O	5-Octadecenal**-**	23.002	0.881
C_16_H_32_O_2_	Palmitic acid P1210	26.623	1.508
C_15_H_30_O_3_	Methyl 2-hydroxy-tetradecanoate	27.418	3.74
C_18_H_36_O_2_	Octadecanoic acid	28.659	0.643
C_19_H_38_O_4_	Hexadecanoic acid,2-hydroxy-1-(hydroxymethyl)ethyl ester	32.4	1.57
C_21_H_42_O_4_	Octadecanoic acid, 2,3-dihydroxypropyl ester	36.032	1.162

**Fig. 7:**
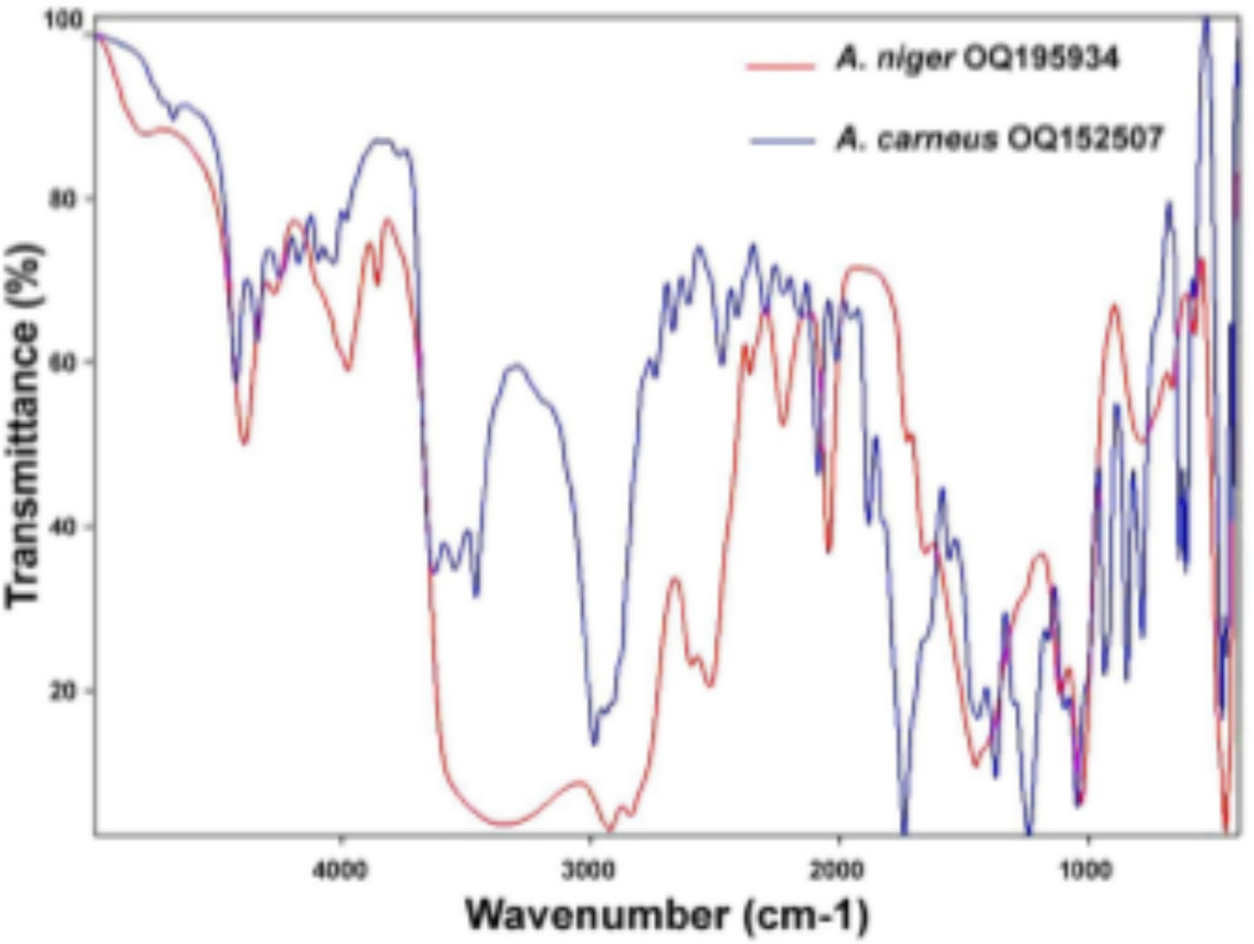
FT-IR spectra of lyophilized biosurfactant produced by *A. carneus* OQ152507 and *A. niger* OQ195934.

FT-IR evaluated the molecular composition of biosurfactants of *A. carneus* OQ152507 and OQ195934 produced under optimal conditions (Fig. 7). The FT-IR spectrum of *A. carneus* surfactants may show that all the compounds are present in the crude *A. carneus* surfactant (Figs. 6 and 7). The characteristic peaks at 3310, 1738, and 1465 cm^− 1^ correspond to the OH, COO, and C = C stretching vibrations, respectively. The IR spectrum of *A. niger* OQ195934 showed the spectrum exhibited characteristic peaks at 3350, 1658, and 1455 cm^− 1^ assigned for OH, COOH, COO, and C = C stretching vibration, respectively (Figs. 6 and 7). Generally, these spectra perfectly match the compound identified through GC-MS analysis. Varjani and Upasani^96^ found that the main classifications for the biosurfactants are glycolipids, fatty acids, lipopeptides, lipoproteins, phospholipids, and polymeric compounds. The current study confirmed that the biosurfactants from *A. carneus* and *A. niger* are classified as glycolipids, which aligns with Bhardwaj et al.^97^ who found that the majority of fungal biosurfactants are lipid derivatives. In the *A. carneus* supernatant, GC-MS showed that palmitic acid ME P1297 (16:0), 9,12-octadecadienoic acid, methyl ester, (E, E)-(19:2), hexadecanoic acid, 2-hydroxy1(hydroxymethyl) ethyl ester (19:0), and octadecanoic acid, 2,3-dihydroxypropyl ester (21:0) were all present. In the *A. niger* filtrate, methyl 2-hydroxy-tetradecanoate (15:0) was the most common acid. In glycolipid biosurfactants, these fatty acid moieties may act as hydrophobic tails. Likewise, the FTIR analysis confirmed that these biosurfactants are glycolipids. Similar results were detected concerning *Mucor hiemalis*, *Mucor indicus*, *Candida glabrata* surfactants, and *Gordonia* sp^88,98^.

## Conclusion

The fungi *A. carneus* OQ152507 and *A. niger* OQ195934, extracted from soil samples tainted with hydrocarbons, exhibited proficient biosurfactant synthesis capacities. The biosurfactant synthesis by these fungi was markedly improved when cultivated under optimum circumstances. The biosurfactants produced by *A. carneus* and *A. niger* demonstrated several advantageous characteristics. These biosurfactants exhibited enhanced emulsification capacity, significantly lowering the surface tension of aqueous solutions. Moreover, they exhibited the capability to augment the extraction of oil from polluted sand and raise the apparent solubility of PAHs. Furthermore, the biosurfactants demonstrated diverse biological activities, including antimicrobial capabilities. Chemical research indicated that the biosurfactants consisted of glycolipid and phospholipid molecules. This study’s findings underscore the potential applications of *A. carneus* and *A. niger* biosurfactants in environmental remediation and industrial approaches.

## Data Availability

Sequence data that support the findings of this study have been deposited in the NCBI with the accession codes of QQ152507 (https://www.ncbi.nlm.nih.gov/nuccore/OQ152507.1/) and QQ195934 (https://www.ncbi.nlm.nih.gov/nuccore/OQ195934).
